# Advanced characterization-informed machine learning framework and quantitative insight to irradiated annular U-10Zr metallic fuels

**DOI:** 10.1038/s41598-023-35619-1

**Published:** 2023-06-30

**Authors:** Fei Xu, Lu Cai, Daniele Salvato, Fidelma Dilemma, Luca Capriotti, Tiankai Yao

**Affiliations:** Idaho National Laboratory, Idaho Falls, ID 83401 USA

**Keywords:** Nanoscale materials, Nuclear energy

## Abstract

U-10Zr Metal fuel is a promising nuclear fuel candidate for next-generation sodium-cooled fast spectrum reactors. Since the Experimental Breeder Reactor-II in the late 1960s, researchers accumulated a considerable amount of experience and knowledge on fuel performance at the engineering scale. However, a mechanistic understanding of fuel microstructure evolution and property degradation during in-reactor irradiation is still missing due to a lack of appropriate tools for rapid fuel microstructure assessment and property prediction based on post irradiation examination. This paper proposed a machine learning enabled workflow, coupled with domain knowledge and large dataset collected from advanced post-irradiation examination microscopies, to provide rapid and quantified assessments of the microstructure in two reactor irradiated prototypical annular metal fuels. Specifically, this paper revealed the distribution of Zr-bearing secondary phases and constitutional redistribution across different radial locations. Additionally, the ratios of seven different microstructures at various locations along the temperature gradient were quantified. Moreover, the distributions of fission gas pores on two types of U-10Zr annular fuels were quantitatively compared.

## Introduction

Machine-learning (ML), a type of artificial intelligence (AI) methods to predict an outcome based on input data, is graining attractions in materials science. ML models have been used to predict the crystal structures and/or properties of materials and build the relationships of material microstructure—processing—property—performance for different material systems^1,2^. ML models have the potential to accelerate the research, development, qualification, and commercial licensing of nuclear materials^3^.

The research and eventually deployment of nuclear materials for commercial use is an expensive and time consuming process. To ensure the function of nuclear component under irradiation at extreme environments (high temperature and/or high pressure and/or highly corrosive environments, etc.), a large number of out-of-pile experiments, in-reactor (irradiation) tests, and post-irradiation examination (PIE) and testing matrix are necessary to achieve a good understanding of degradation mechanism and predictable material performance during service^4^. The goal of present study is to use ML models to analyze a large number of electron microscopy images and spectrum datasets to facilitate the developing of  mechanistic understanding the irradiation induced microstructure and phase change of U-10wt% Zr (U-10Zr) fuel, a primary candidate for next generation sodium cooled fast reactors (SFRs)^5^. Our long term goal is to accelerate fuel qualification and licensing for commercial use.

U-10Zr fuels were tested rigorously in test reactors, such as Experimental Breeder Reactor II (EBR-II) and Fast Flux Test Facility (FFTF) from the 1960s to the 1990s^6,7^. Advanced PIE data at the sub-nanometer to micrometer scale has been recently collected and analyzed ^8–10^. Two most fuel performance related phenomena of this fuel type are fuel-cladding chemical interaction (FCCI) ^11^and fuel constitutional redistribution (in this paper, we are focusing on Zr redistribution)^12^

FCCI is a chemical reaction between nuclear fuel and cladding (a thin-walled metal as first barrier for retention of fission products and actinides), which creates an important challenge to overcome for metallic fuels^7,13^. FCCI is generally dominated by the reactions between lanthanide fission products and iron-based cladding, resulting in eroded cladding with deteriorated mechanical properties, which adversely impacts the fuel integrity and performance. Lanthanides are transported from the hottest fuel center to the cold inner cladding through interconnected pores filled by fission gas, with the help from liquid Cs (a fission product with melting temperature of ~28 °C) and sodium if present in case of solid U-10Zr^9,14^. However, porosity distribution regarding lanthanide movement in irradiated U-10Zr remains obscure,  making it difficulty understand lanthanide transportation mechanism and FCCI mitigation.

In addition, a clear and quantitative understanding of pore characteristics (distribution, size, etc.) can help better predict the fuel property degradation. For example, fission gas pores can lead to a 35% reduction of thermal conductivity for metallic fuel^15^. Fission gas pores show different sizes and distribution patterns along a radial thermal gradient inside irradiated U. The gas pores are primarily round and significantly smaller in the hot center zone than the ones near the cladding zone^16^. How thermal conductivity will be impacted remains poorly understood.

Another key factor is constitutional redistribution. Fuel constituent radial redistribution during irradiation changes the local fuel composition and therefore performance critical properties, such as melting temperature^8^. The temperature gradient within the U-10Zr fuel, as well as irradiation enhanced diffusion, causes zirconium and uranium to migrate in the opposite directions, resulting in different zones with different characteristic microstructures and crystal structures. The zirconium redistribution can be beneficial, because it can increase the solidus temperature in the hot fuel center where Zr migrates to^6^. However, the zirconium redistribution may also have adverse impacts on the fuel performance as it may influence/degrade the material properties (e.g., thermal conductivity) in the rest of the fuel^7,10^. It is essential to achieve a comprehensive and quantified understanding of the zirconium redistribution (phase distribution) in order to better predict fuel performance^11^. A U-10Zr fuel cross-section would host Zr redistribution in three major phases: $\upgamma$ phase (a continuous body centered cubic solid solution between U and Zr), noted as the (U, Zr) matrix; $\mathrm{\alpha }$-U matrix; and $\mathrm{\alpha }$-U + UZr_2._ The Zr redistributes across the pin radius and forms up to three distinctive zones by different U/Zr contents^17^.

**Figure 1:**
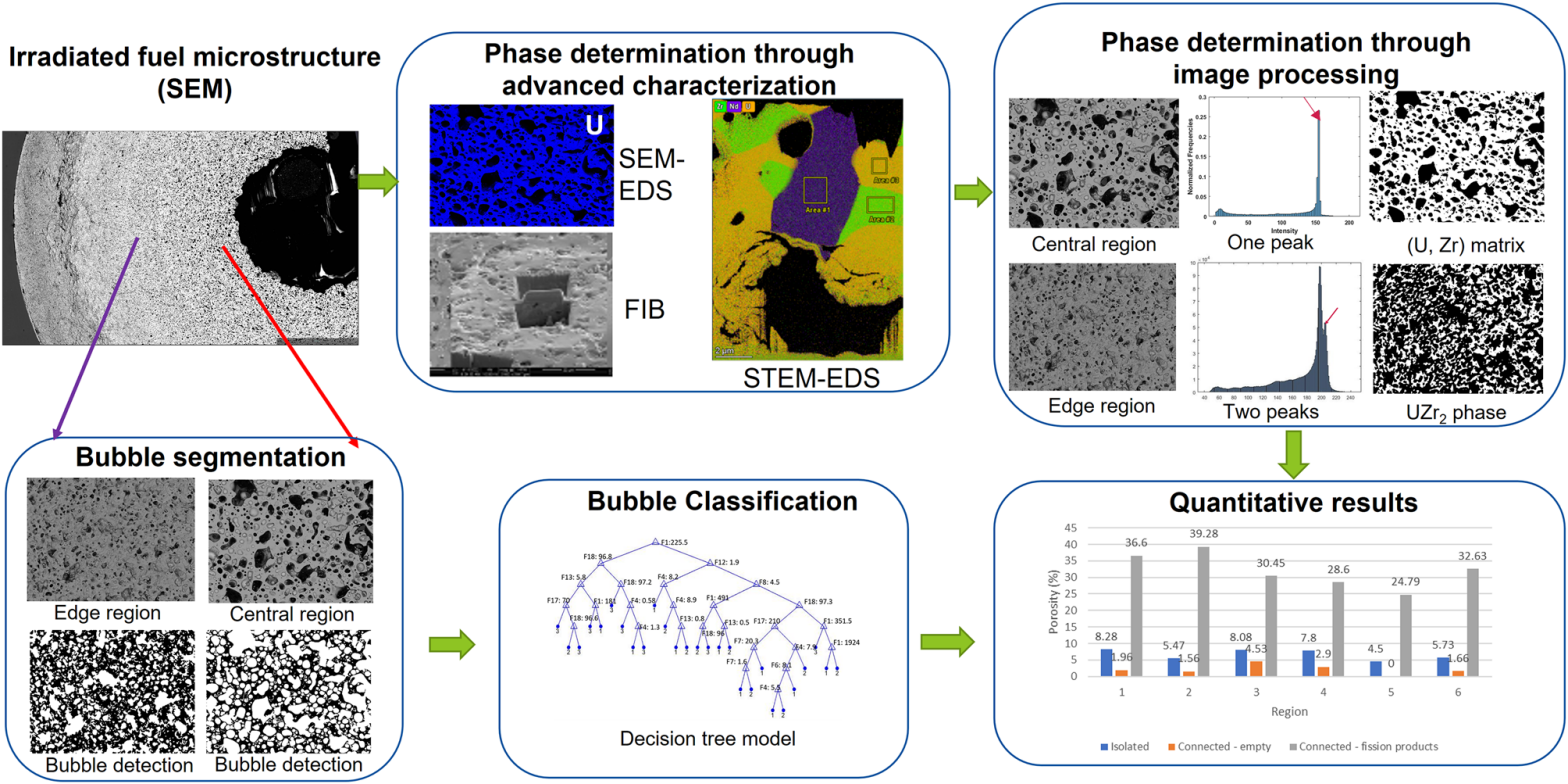
Workflow of the proposed advanced characterization informed ML modeling enhanced post irradiation examination method on AF2.

In our previous study, machine learning (ML) model has been successfully applied to characterize and classify pores and gained quantitative insights into the lanthanide transportation of an U-10Zr annular fuel (AF1)^17^. In this paper, another annular U-10Zr fuel, named AF2 was investigated. The pipeline of the proposed method is shown in Fig. 1. Two major processes were involved in this study: (1) analyzing the distributions of two major elements U and Zr and (2) exploring the fission gas pore distribution in the fuel microstructure from hot region to colder region close to cladding. In the process of fission gas pore analysis, the UZr_2_ phase is identified; then pores are segmented from the background (detailed in “Phase identification and analysis” and “Pore detection and classification” sections); and then a trained Decision Tree classifier is applied to cluster the pores into categories such as large/intermediate/small size and connected/isolated (as described in “Pore statistics and its implication on nuclear fuel performance” section). Lastly, the quantitative results of the new advanced fuel are generated and used to obtain conclusive findings by comparing the two advanced fuels.

## Experiments and models

### Experimental data

**Figure 2:**
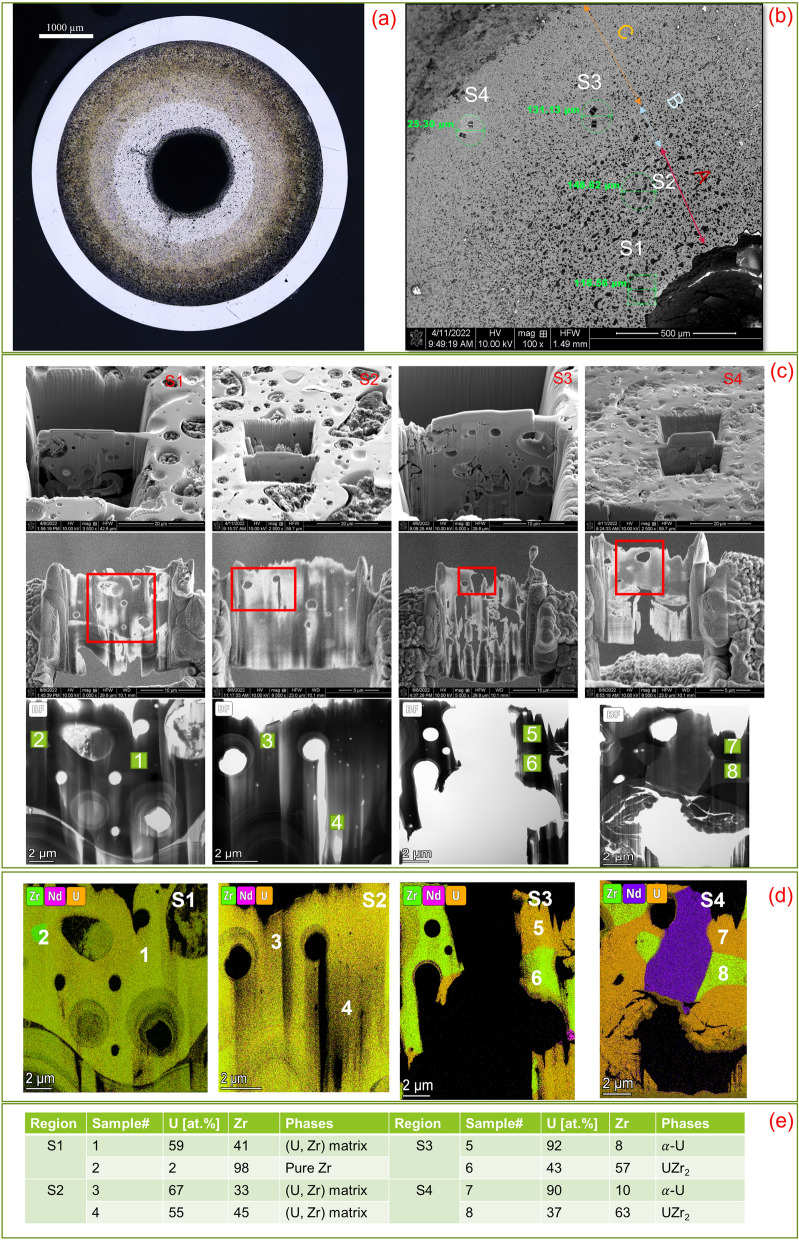
The micrographic cross-section of AF2 with 4.3% fission per initial metal atom (FIMA) burnup (**a**); and three significant regions of the cross-section and four selected locations for STEM characterization (**b**); STEM compositions of elements study at different locations on the fuel with 8 selected phases (**c**,**d**). The corresponding phase composition of U and Zr for each phase (**e**). Microstructure UZr_2_ is indicated as phases 6 and 8 in locations S3 and S4.

Idaho National Laboratory (INL) has been the leading national laboratory for research and development (R&D) of metallic fuel. Advanced characterization techniques, e.g., focused ion beam (FIB) sampling and scanning transmission electron microscopy (STEM) characterization, have been applied to investigate U-10Zr fuel samples irradiated in EBR-II and FFTF to gain thorough understanding of fuel microstructures and property evolutions as a function of irradiation burn up. STEM Energy dispersive spectroscopy (EDS) and TEM images of fuel samples were used to analyze element composition and identify new phases. We used scanning electron microscopy (SEM) EDS images for the distributions of chemical elements, and STEM EDS data for more accurate chemical composition for each microstructure. The STEM EDS and selective area electron diffraction patterns (SAED) have successfully identified different phases (crystal structures and compositions) within irradiated U-10Zr^8^ in the nano-meter scale (see Fig. 2).

**Figure 3:**
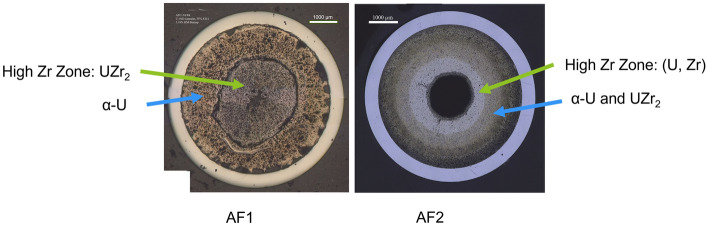
The micrographic cross-sections of two U-10Zr annular fuels.

The purpose of this study was to quantitatively understand the Zr redistributions and microstructure features of two irradiated annular U-10Zr fuels (AF1 and AF2). AF1 and AF2 were manufactured using a similar process except that there was an extra fabrication step for AF2^18^. The AF2 fuel slug was machined after casting to create a small (< 25 µm) well-controlled gap between the fuel and cladding. However, the AF1 fuel slug was placed in the pin without this machining step and resulted in a significant gap (> 50 um) between the fuel slug and the cladding. In addition, the irradiation capsules containing AF1 had defects that could result in local variations in temperature, which eventually caused the early termination of the irradiation experiment^19^. The increased gap as well as manufacturing defects caused irregularities on fuel-cladding contact with large voids. Because of helium bonded instead of sodium bonding, the large fission-gas filled voids between fuel and cladding result in significantly higher fuel temperatures during irradiation. Based on BISON calculation, the peak fuel temperature can be 100 °C higher if small voids present on the fuel-cladding interface of AF1 compared to the nominal case where the fuel is fully bonded with the cladding^20^. On the other hand, the irradiation temperatures experienced by AF2 were more reasonable^18^. The irradiation condition of the two advanced annular U-10Zr fuels is shown in Table 1 and the optical cross-section images of the two fuels are shown side-by-side in Fig. 3. (U, Zr) matrix exists on the center zones of both fuels. In the outside of (U, Zr) region, AF1 is $\mathrm{\alpha }$-U matrix, while AF2 is $\mathrm{\alpha }$-U matrix with UZr_2_ secondary phase. Due to polish, the fuel regions with radius greater than 1.7 mm from fuel center are removed and therefore not investigated in this study. Seven types of features exist on the fuel microstructure: 1) four phases, $\mathrm{\alpha }$-Uranium matrix ($\mathrm{\alpha }$-U), U rich with Zirconium (Zr) matrix phase, noted as (U, Zr) matrix, pure Zr, Zr rich with U, noted as UZr_2_; and 2) three types of pores (isolated pore, connected empty pore, and connected pore with fission products inside). Due to the limitation of the 2-dimensional images and the cutting location, some fission products inside the pores may not be observed. In future, the 3D microstructure from X-ray tomography as well as focused-ion-beam sections will be investigated to couple with SEM images. As shown in Fig. 2a,b, the investigated fuel cross-section could be separated into three concentric zones with different compositions of elements. Three types of pores are major microstructural features in the (U, Zr) matrix Zone A. Three types of pores, (U, Zr) matrix and phase UZr_2,_ consist in Zone B. Zone C mainly contain pores and UZr_2_ in the $\mathrm{\alpha }$-U matrix. EDS results provided the elemental mapping of U, Zr, and fission products.

**Table 1:** Information of the two advanced U-10Zr fuels.

Fuel ID	Alloy	Fuel form	Bond material	Nominal smear density (%)	Burnup (FIMA, %)	Cladding temperature	FCCI
AF1	U-10Zr	Annular	Helium	55	3.3	540–600 °C	High
AF2	U-10Zr	Annular	Helium	55	4.3	600 °C	Low

As shown in Fig. 2c,d, multiple phases were present even in nano-meter scale. The EDS in scanning electron microscope (SEM) can provide elemental mapping in the micron-scaled area, but it is impossible to derive the phase information from pure SEM-EDS. It is a challenge to bridge this phase information in a nano-meter scaled area from STEM, with the micron-scaled information from SEM-EDS, to obtain more meaningful statistical insights. In this study, EDS images were collected at six representative locations for a single fuel cross-section (see Fig. 7a). Additionally, STEM images from four locations were collected to identify the phases (see Fig. 2c,d,e). Moreover, a partial cross-section image is generated by stitching 587 high resolutions Back Scattered Electron (BSE) images which were collected using a JEOL JSM-7000F SEM with a 20 keV accelerated electron beam. The images have pixel sizes of 0.05 µm/pixel. In “Phase identification and analysis” section, image processing techniques have been used to identify phases from EDS mapping with the aid from TEM diffraction pattern results.

**Figure 4:**
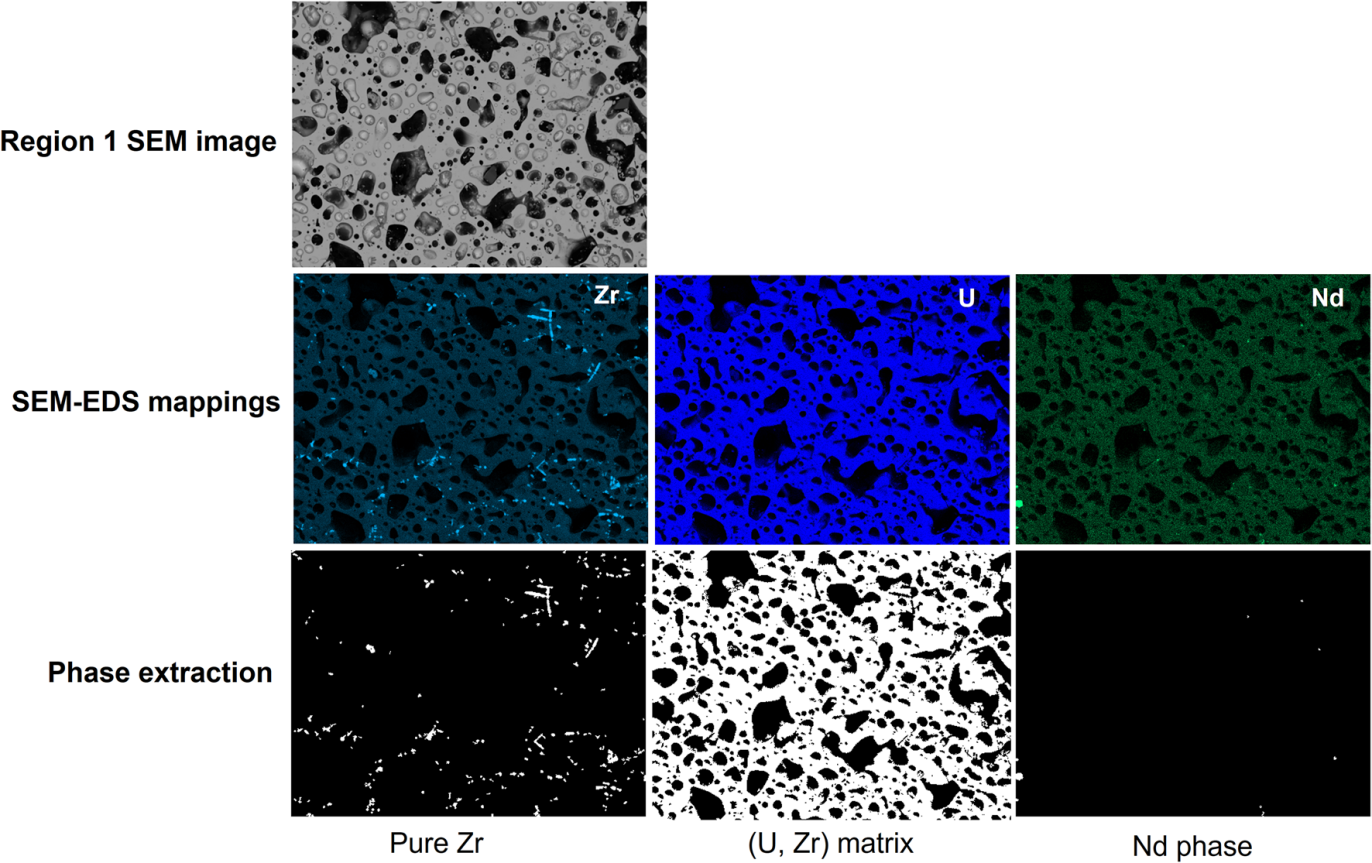
Region 1 of AF2 (close to the hot fuel center) annotated Pure Zr, U, and Nd images. Location with red arrow: pure zirconium (> 90% Zr, < 10% U); orange arrow: pores.

**Figure 5:**
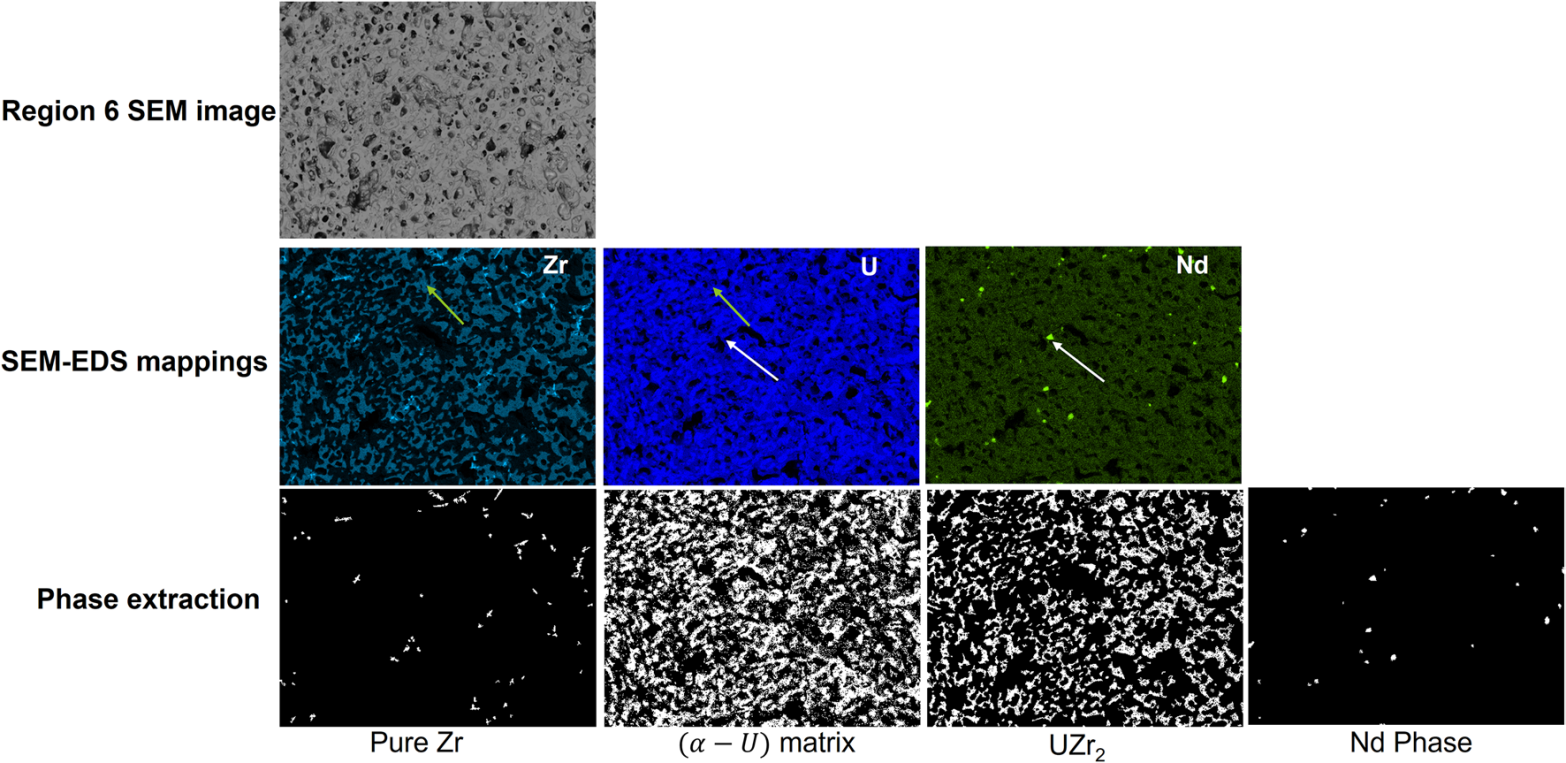
Region 6 of AF2 annotated Pure Zr, α-U matrix, UZr_2,_ and Nd images. green arrow: UZr_2_ (> 50% Zr, < 45% U); white arrow: Nd.

Figures 4 and 5 show two examples of SEM-EDS images. The original SEM-EDS images used the RGB color model, and the color brightness has a linear relationship with the concentration of elements, (i.e., the brighter a pixel is, the higher concentration an element has). Figure 4 shows the ‘Region 1’ SEM image patch and its corresponding three SEM-EDS images (Zr, U and Nd). Figure 5 is the images of ‘Region 6.’ By using SEM-EDS images, this study was able to generate partially annotated gas pores, different types of microstructures, and calculate the statistics.

### Phase identification and analysis

As shown in Figs. 4 and 5, the original SEM image is grayscale. In digit image, the smallest element is noted as pixel. Each pixel has its intensity, and higher intensity appears brighter in the image. In grayscale image, the intensity in the image is between [0, 255]. The pure Zr, UZr_2_ and Nd phases can be easily picked up from SEM-EDS images (refer to the colored arrows in Figs. 4 and 5). The pure Zr, pure Nd, (U, Zr) matrix, and α-U matrix are defined as the pixels with relative higher intensities in the corresponding SEM-EDS mapping images. The phase UZr_2_ is defined as moderate intensities in both Zr and U images. The pores appear relatively dark in SEM images. As discussed in^19^, advanced STEM-EDS characterization shows pure Zr is assumed to be more than 90% Zr, alpha-U more than 80% Uranium, and high Nd more than 50% Neodymium in the composition. A multiple threshold segmentation method helps in separating the pixels into different groups according to their intensity levels, but cannot tell the chemical compositon of the group. Therefore, we combine the STEM-EDS characterization results together to generate the initial annotated images of pure Zr, (U, Zr) matrix, $\mathrm{\alpha }$-U matrix, UZr_2_, Nd, and pores. ${\mathrm{T}}_{\mathrm{k}1}\sim {\mathrm{T}}_{\mathrm{k}3}$ (k can be Zr, U, or Nd) are the threshold values generated by the method on Zr, U and Nd from SEM-EDS images. Since different percentages of Zr exist in the four different microstructures/phases, such as pores with 0% Zr, (U, Zr) matrix or ${\upalpha}\text{-U}$ matrix with Zr content less than 40%, UZr_2_ with 50–80% Zr, and pure Zr with over 90% Zr, we applied the multi-thresh method with 3 threshold values to segment the Zr SEM-EDS image into 4 groups, eventually map into the four microstructures/phases by combining the STEM-EDS Zr content range for different microstructures. From Eq. (1), the (U, Zr) matrix or ${\upalpha}\text{-U}$ matrix cannot be distinguished only based on SEM-EDS of element Zr, thus, we need to combine with Eq. (2) which segments out the ${\upalpha}\text{-U}$ matrix first. If ${\upalpha}\text{-U}$ matrix is not detected from Eq. (2), the other condition of Eq. (1) will obtain the (U, Zr) matrix, verse vise. Uranium and Nd are consisting of two distinguish microstructures/phases respectively, high U (${\upalpha}\text{-U}$), Nd and pores. Therefore, only one threshold value is needed in Eqs. (2) and (3). For instance, in Zr SEM-EDS image, the pixels are separated into four groups based on the intensities as shown in Eq. (1).

1 $${\mathrm{G}}_{\mathrm{Zr}}\left(\mathrm{i},\mathrm{j}\right)= \left\{\begin{array}{ll} \mathrm{Pure \; Zr}, & \quad \mathrm{ if } \; {\mathrm{I}}_{\mathrm{Zr}}\left(\mathrm{i},\mathrm{j}\right)\ge {\mathrm{max}(\mathrm{T}}_{\mathrm{zr}1},{\updelta }_{1}*255)\\ \mathrm{No \; Zr \; element},& \quad \mathrm{ if} \; {\mathrm{ I}}_{\mathrm{Zr}}\left(\mathrm{i},\mathrm{j}\right) < {\mathrm{T}}_{\mathrm{zr}2}\\ {\mathrm{UZr}}_{2}, & \quad \mathrm{ if } \; {\mathrm{I}}_{\mathrm{Zr}}\left(\mathrm{i},\mathrm{j}\right) > {\mathrm{T}}_{\mathrm{zr}3}\mathrm{ and }{\mathrm{I}}_{\mathrm{Zr}}\left(\mathrm{i},\mathrm{j}\right)< {\mathrm{max}(\mathrm{T}}_{\mathrm{zr}1},{\updelta }_{1}*255) \\ \left(\mathrm{U},\mathrm{ Zr}\right)\mathrm{ matrix \; or \; \alpha{-}U \; matrix}, & \quad \mathrm{others}\end{array}\right.$$

2 $${\mathrm{G}}_{\mathrm{U}}\left(\mathrm{i},\mathrm{j}\right)= \left\{\begin{array}{ll} \mathrm{High \; U}, & \quad \mathrm{ if } \; {\mathrm{I}}_{\mathrm{U}}\left(\mathrm{i},\mathrm{j}\right)\ge {\mathrm{max}(\mathrm{T}}_{\mathrm{u}1},{\updelta }_{2}*255)\\ \mathrm{No \; U \; element}, & \quad \mathrm{ if} \; {\mathrm{ I}}_{\mathrm{U}}\left(\mathrm{i},\mathrm{j}\right) < {\mathrm{T}}_{\mathrm{u}2}\end{array}\right.$$

3 $${\mathrm{G}}_{\mathrm{Nd}}\left(\mathrm{i},\mathrm{j}\right)= \left\{\begin{array}{ll} \mathrm{High \; Nd}, & \quad \mathrm{ if } \; {\mathrm{I}}_{\mathrm{Nd}}\left(\mathrm{i},\mathrm{j}\right)\ge {\mathrm{max}(\mathrm{T}}_{\mathrm{nd}1},{\updelta }_{3}*255) \\ \mathrm{No \; Nd \; element}, & \quad \mathrm{ if} \; {\mathrm{ I}}_{\mathrm{Nd}}\left(\mathrm{i},\mathrm{j}\right) < {\mathrm{T}}_{\mathrm{nd}2}\end{array}\right.$$

4 $$\mathrm{Pores}=\mathrm{ No \; Zr}, \mathrm{ U},\mathrm{ or \; Nd \; in \; corresponding}$$

where: ${\mathrm{I}}_{\mathrm{k}}$ is the corresponding SEM-EDS image; ${\mathrm{G}}_{\mathrm{k}}$ is the corresponding segmentation result; and k can be Zr, U, or Nd.

To match with the STEM-EDS characterization, we set ${\updelta }_{1}$ as 0.9, ${\updelta }_{2}$ as 0.8, and ${\updelta }_{3}$ as 0.4 in Eqs. (1)–(4). The threshold values are utilized to segment the image regions into different groups, and the parameters ${\updelta }_{1}\sim {\updelta }_{3}$ are mapping the groups into corresponding microstructures. The annotated images were generated only based on SEM-EDS images (Fig. 4 for Region 1 and Fig. 5 for Region 6). The distributions of six types of compositions based on analyzing the SEM-EDS images of six different locations on the fuel are shown in Table 2. The values of Zr concentrations are calculated based on Eq. (5). The number 0.45, 0.6, 0.98 and 0.1 are the at.% of Zr determined by STEM-EDS in the different compositions as shown in Fig. 2d:

**Table 2:** Distributions of five types of compositions and porosities on AF2.

Zone	Region*	Distance from cladding (mm)	Radius (mm)	$\alpha{\text{-}}U$	Nd	Pure Zr	UZr_2_	(U, Zr)	Porosity	Zr conc. (at.%)
A	1	1.7	0.69	0.00%	0.04%	1.29%	0.00%	71.66%	27.04%	33.51%
	2	1.6	0.81	0.00%	0.34%	1.23%	0.00%	69.48%	29.18%	32.47%
	3	1.5	0.89	0.00%	0.32%	1.18%	0.00%	76.73%	22.02%	35.68%
	4	1.4	0.97	0.00%	0.65%	1.12%	0.00%	76.53%	22.20%	35.54%
C	5	0.8	1.6	55.40%	0.00%	0.70%	35.86%	0.00%	8.04%	27.74%
	6	0.7	1.68	57.59%	0.12%	0.07%	36.51%	0.00%	5.71%	27.73%

5 $$Zr concentration={P}_{\left(U,Zr\right)} \times 0.45+ {P}_{{UZr}_{2}} \times 0.6+{P}_{\left(Pure Zr\right)} \times 0.98+ {P}_{\left(\alpha -U\right)} \times 0.1$$

**Figure 6:**
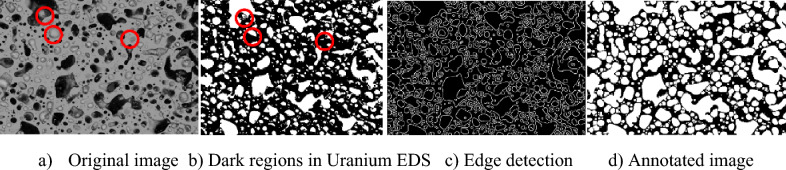
Annotated image generation on AF2. (**a**) Original image. (**b**) Dark regions in Uranium SEM-EDS. (**c**) Edge detection. (**d**) Annotated image.

Three experts were involved in determining the annotated results. The multi-threshold method generated accurate results on the phases pure Zr, high Nd, and UZr_2_. However, due to the height or angle factors when collecting the SEM-EDS images, some pores are not visible in the SEM-EDS images, as shown in Fig. 6a,b marked with red circles.

**Figure 7:**
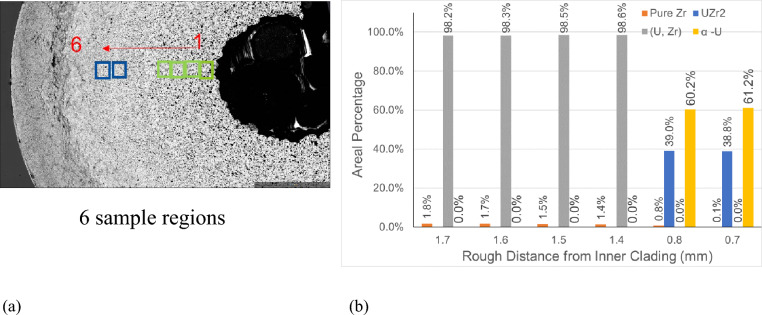
(**a**) The illustration of the six locations on AF2 where the EDS were collected. (**b**) The phase information at the six locations. Note: The y-axis of (**b**) is the areal percentage from the EDS and SEM. In the statistic result, the regions with other elements or pores were counted to the matrix, either (U, Zr) or α-U.

To verify and solve the problem, an edge detection method was employed to the original SEM image of the Region 1 first as shown in Fig. 6c, then combining the EDS results, the experts manually labeled the pores by drawing the boundaries and filling them to generate the final annotated image as shown in Fig. 6d. The porosity of the annotated result of Region 1 is around 50.3%, which was distinct from approximately 27% in the 1st row, second last column of Table 2. The verification results revealed EDS-SEM measurements inside the pores may be incorrect since these images reflected the lower surface element information under the pores. The annotated image helps demonstrate the distributions of microstructures along the cross-section from hot region to cladding regions. Additional results are shown in Fig. 7. Figure 7a illustrates the six sample locations where EDS measurements were collected along the fuel cross-section. Sample regions 1–4 are in Zone A of Fig. 2b and Sample regions 5 and 6 are in Zone C of Fig. 2b.

Figure 7b shows the relative areal percentage of four phases, pure Zr, UZr_2_, (U, Zr) matrix, and α-U. The sample regions 1–4 contain < 2% pure Zr and about 98% (U, Zr) matrix. The pure Zr content increases slightly towards the fuel center, from 1.4 to 1.8%. The pure Zr phase was precipitated from (U, Zr) phase during the cooling after irradiation, while at the irradiation temperature, the (U, Zr) phase was supersaturated^8^. The increased pure Zr phase towards the fuel center from regions 1–4 is consistent with the increase of Zr concentration observed in previous study^21^. Sample regions 5 and 6 are consistent and contain about 39% UZr_2_ and 61% α-U, while in the AF1, UZr_2_ phase formed in the fuel center, and α-U matrix formed in the region close to the cladding^8,22^. There is small amount (0.8% and 0.1%) of pure Zr at regions 5 and 6. Similarly, the Zr precipitation was also observed in the α-U region of AF1^8^. Though Zr tends to migrate to central high temperature region, the Zr low mobility in α-U limits its diffusion in the fuel peripheral region^21^. The fuel peripheral region contains α-U and δ-UZr_2_ when the outer fuel temperature lower than 617 °C^10^. The presence of UZr_2_ created new interfaces that can stop the movement of the lanthanides^22^, so the appearance of UZr_2_ close to the cladding may serve as a barrier for lanthanide transportation to reach cladding, which is another possible reason why AF2 has less FCCI.

### Pore detection and classification

Xu, et al. proposed a workflow to detect the pores and then classify the pores into three categories: connected pores with fission products, connected empty pores, and isolated pores^23^. The method mainly contained four steps: detect the darker and brighter pores using two different thresholds; segment the pores’ boundary using morphological operators; develop a decision tree model to classify the pores into three categories based on approximately 800 manually annotated pores; and divide the cross-section into 11 rings and generate the distributions based on pores’ different properties^17^.

**Figure 8:**
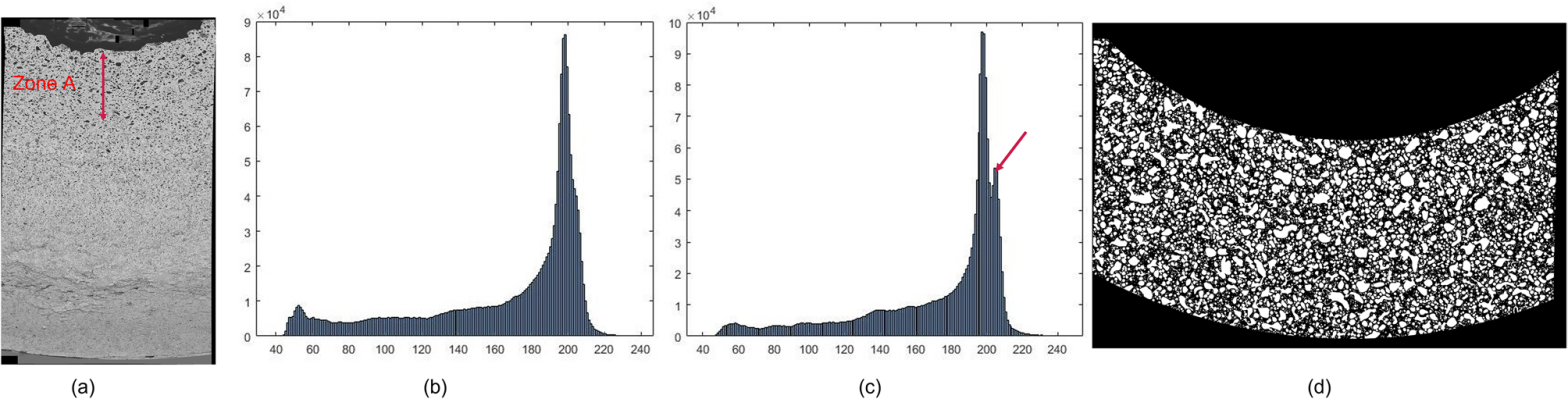
Background removal result on AF2. (**a**) Partial cross-section, (**b**) intensity histogram without UZr_2_ phase (**c**) with UZr_2_ phase(d) pore detection in Zone A.

In this paper, a partial cross-section stitched image with image size 12,619 × 18,401 pixels with 0.05 µm/pixel was generated, shown in Fig. 8a. A full fuel cross-section, with radius 48,633 pixels and image size 97,852 × 97,266 pixels, was created by calculating the center and radius of the cross-section based on the cladding edge. Since the center is outside the partial image as shown in Fig. 8a, we generate a mask for Zone A which only counts the partial cross-section, and the radius is from 0.665 to 0.985 cm. As shown in Fig. 2b, the fuel cross-section could be separated into three regions with different compositions of microstructures. Obviously, the composition of UZr_2_ is the key to identifying different areas. Based on the observation, (α-U) matrix appears much brighter (U, Zr) matrix on SEM images, and the intensities of regions in (α-U) matrix are over 200. UZr_2_ is the secondary phase forming from (α-U) matrix. The histogram of the SEM images shows the frequencies of corresponding intensities. The intensity of the peak frequency in the histogram potentially indicates the matrix information. Regions (1–4) have similar histogram curve as Fig. 8b, however a second peak frequency appears in the histograms of Region 5 and Region 6, as shown the red arrow in Fig. 8c, which indicate the new phases/matrix existing with much brighter appearances and higher intensity values on SEM images. The more Uranium, the higher the frequency value of the histogram peak. To compare the distributions in^17^, we generated the area A with a radius range [665 µm, 1065 µm] from the fuel center and detected the pores using the method in^17^. The result is shown in Fig. 8d with more than 17,700 pores.

**Figure 9:**
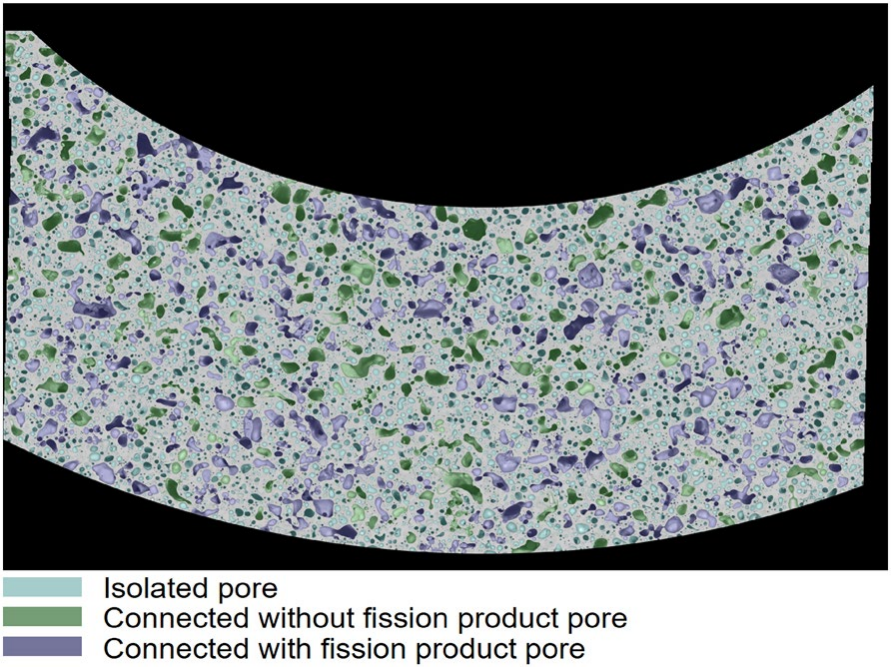
Pore classification of Zone A on AF2. The spatial resolution is 0.05 µm/pixel.

We developed a decision tree model^17^ to classify the pores into three types by extracting 18 features, including the pore size, intensity histogram, mean intensity, the standard deviation of intensity, intensity range, and the shape convexity. In this paper, we applied the same model to the pore detection result of Zone A, shown in Fig. 9. As discussed in literature^17^, the model obtained good overall performance, especially on the isolated bubbles. However, some of the connected bubbles were misclassified to isolated bubbles since the decision tree model trained with unbalanced limited data, only including very small number of connected bubbles. We will annotate more data for training and develop new models to obtain better bubble segmentation and classification performance in the future.

### Pore statistics and its implication on nuclear fuel performance

The effect of fission gas pores on nuclear fuel performance has been focused on its impact on fuel swelling^6^. The fission product atoms have a limited solubility inside fuel matrix and tend to precipitate out and cluster into pore form. The pores can be either intragranular (within grains) or intergranular (on grain boundaries). With increase of fuel burn up and gaseous fission products, both intragranular and intergranular pores can grow to larger sizes through fission gas diffusion. The large intergranular pores can eventually become connect and act as short pathway for fission gas releasing into the plenum inside the fuel rod. The intragranular pores may also grow large enough to link, causing additional fuel swelling^24^. Because both intergranular and intergranular pores can cause fuel swelling, in this study, pores are not distinguished between intragranular or intergranular, but rather categorized based on their morphological characteristics (size, interconnected/isolated, and whether containing fission products (lanthanides)).

**Figure 10:**
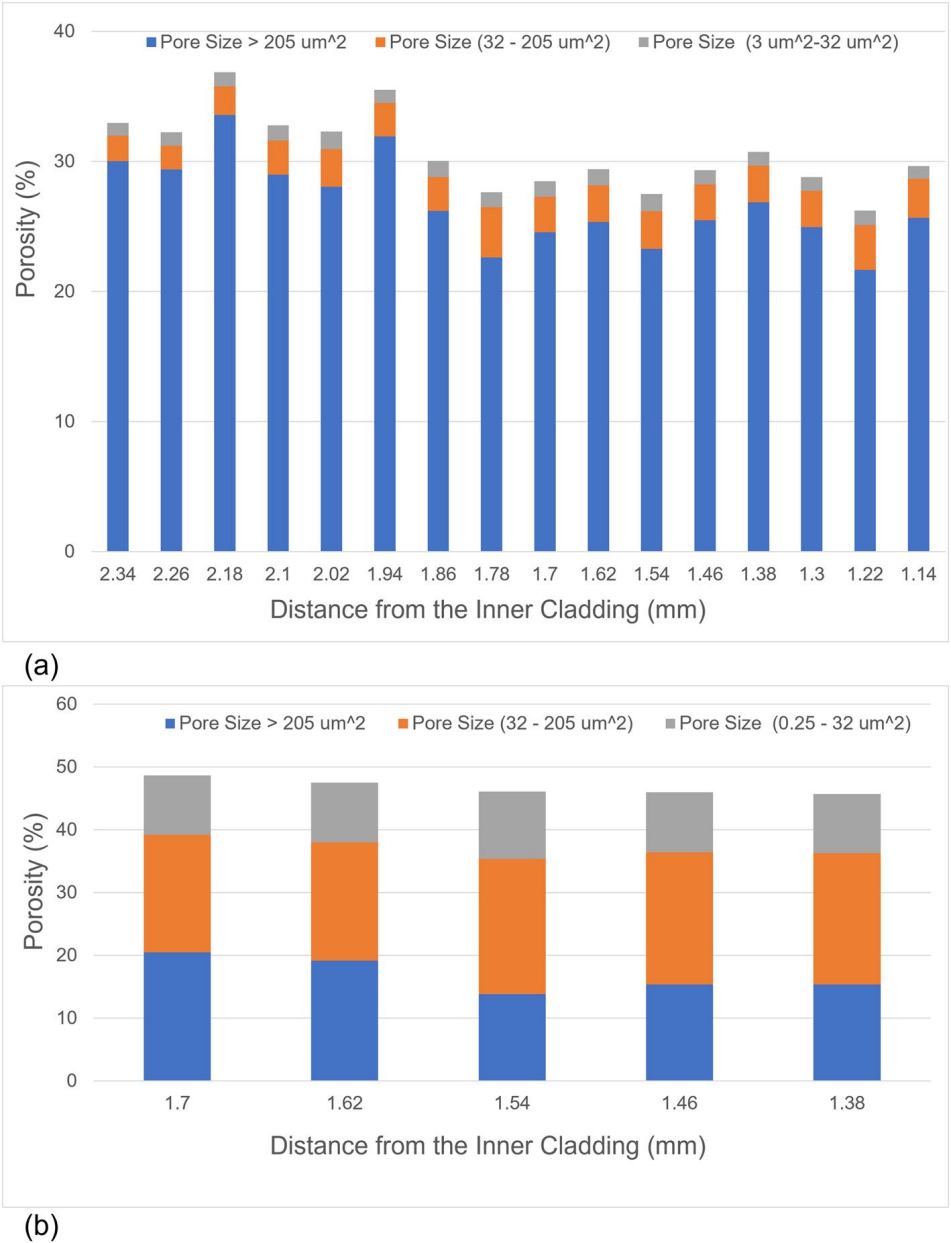
The porosity and contribution from different sized pores of AF1 (**a**) and AF2 (**b**) as a function of distance from the inner cladding. Both are in the high Zr zone of AF1 and AF2 as shown in Fig. 3.

**Figure 11:**
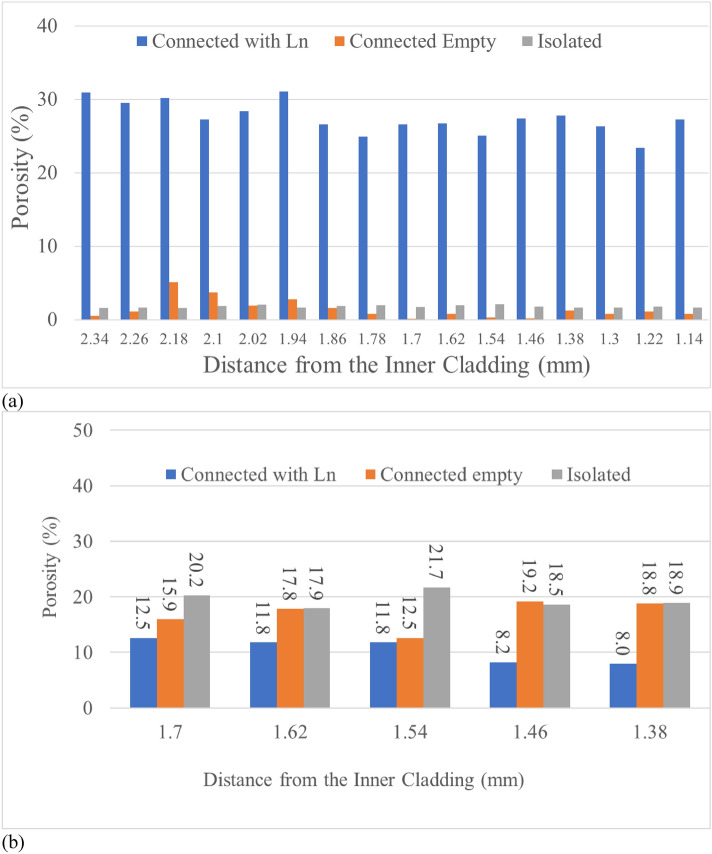
The porosity contribution by three different pore types as a function of distance from the inner cladding of the two fuels. (**a**) AF1, (**b**) AF2.

Accurate pore statistics is the first step to derive the effect of gas pores on fuel performance. The pore statistics of a total of approximately 50,000 pores over an area of 20 mm^2^ for the AF1 U-10Zr fuel and approximately 17,000 pores over an area of 0.4 mm^2^ for the AF2 U-10Zr fuel, are shown in Figs. 10 and 11. We use the equivalent circular diameter (ECD) to measure the size of the pores. The pores are separated into large pores (area coverage > 205 µm^2^, corresponding to ECD of 16.2 µm calculated by  $\mathrm{diameter}=2\times \sqrt{\mathrm{area}/\uppi }$), the intermediate pores (32 < area ≤ 205 µm^2^, or ECD 6.4 < ECD ≤ 16.2 µm of round-shaped pores), and small pores (area ≤ 32 µm^2^, or ECD ≤ 6.4 µm of round-shaped pores). On the other hand, the pores are classified into three categories based on the pore interconnection: isolated pore, connected without fission products, and connected with fission products.

Figure 10a,b are an overview of the porosity and contribution by pores from different categories on the fuel cross section for AF1 and AF2 fuels, respectively. Because of different magnification of the SEM images taken for AF1 and AF2, the minimum identified pore size at AF1 is 3 µm^2^ while it is 0.25 µm^2^ at AF2, which can contribute to a very small portion of porosity at AF2 but are not detectable at AF1. As mentioned in “Phase identification and analysis” section, the pores of the imaged area in Fig. 6 are manually identified and the calculated porosity is 50.3%. This value matches well with Fig. 10b where the fuel central region of AF2 has a porosity close to 50%. The AF1 and AF2 fuels have very different microstructural features. In the high Zr region of the two fuels (fuel center), AF2 has higher porosity than AF1 (nearly 45% to 50% porosity in AF2, 25% to 40% porosity in AF1). Because the central void of AF1 is more filled than AF2, it is expected that AF1 should have higher overall porosity than AF2. Note here only the porosity in the high Zr region (fuel central region) is compared. Local porosity in the high Zr-region (fuel central region) is higher in AF2 than that in AF1. Another finding is that large pores in AF1 contributes more than 85% of the porosity, while AF2 is dominated by small and medium size pores and the number of large pores on AF2 is less than 20%.

In addition, AF2 has more isolated and connected without lanthanides pores as shown in Fig. 11b, while the AF1 has much more connected pores with lanthanides inside as shown in Fig. 11a. These statistical data matches well with the observations that AF2 has much less FCCI, because connected pores are envisioned as the pathway for lanthanides transported from fuel center to the cladding along temperature gradient. The small or isolated pores contribute minimal to the lanthanide movement or may even limit these movement, therefore, there are much fewer pores with lanthanides for AF2 as well as fewer lanthanides transported through the interconnected pores (shortcut) to the inner cladding for FCCI.

Due to the lack of annotated data, the porosity analysis for other image patches was not validated; Also, microstructure analysis based on image processing techniques from SEM-EDS data cannot be directly applied to the entire cross-section due to the limitation of time and labor for a whole cross section SEM-EDS. Our future work focus on building a benchmark of pores and microstructures on advanced U-10Zr fuels to accelerate the PIE of irradiated fuel. For this purpose, more accurate and efficient machine learning models are needed to detect and classify the pores and microstructures to better support quantitative analysis on fuel performance.

## Conclusion

This work developed a ML modeling workflow that integrates multi-scaled microscopic images (from millimeter to nanometer) from multiple sources (SEM, STEM images and EDS) to enhance the post irradiation examination of reactor irradiated prototypical annular U-10Zr metallic fuel. To help develop mechanistic understandings for fuel performance, this work provided quantitative results on the distribution of ⍺-U and UZr_2_ phase as well as the detection and classification of pores.

The Following conclusions can be drawn:

1. Phase detection model is developed by integrating SEM image, SEM–EDS, and STEM-EDS data. To our knowledge, this is the first time the cross link of materials characterization methods is used for ML model development.
2. Both annular fuels have high Zr region in the center. However, AF2 shows a region with mixed phase of ⍺-U and UZr_2_, which is not observed in AF1.
3. The porosity of high Zr region in AF2 is close to 45–50%, which is much higher than that of AF1 (25–40%).
4. More than 85% porosity belongs to the large pores in AF1, while 80% of porosity of AF2 is small and medium size pores.
5. Developed ML models are highly transferable for pore detection and classification in irradiated metallic fuel. 

Quantitatively assessment of Zr concentration s and porosity as well as pore detection and classification along the thermal gradient from fuel center region to cladding of irradiated metallic fuel is beyond human’s capability. The new framework accelerates the quantitative analysis and will serve as bridges between PIE observation and fuel performance modeling efforts which will ultimately accelerate fuel qualification.

## Data Availability

The datasets generated and/or analyzed during the current study are not publicly available due to the laboratory policy but are available from the corresponding author on reasonable request.
